# Supplementary material for: Bayesian polynomial neural networks and polynomial neural ordinary differential equations
Source: PLoS Comput Biol. 2024 Oct 10;20(10):e1012414. doi: 10.1371/journal.pcbi.1012414 (PMC11476690; doi:10.1371/journal.pcbi.1012414)
Supplement: S1 Text — (PDF) [file pcbi.1012414.s001.pdf]

Bayesian polynomial neural networks and polynomial neural ordinary differential equations

Colby Fronk[1*], Jaewoong Yun[2,3], Prashant Singh[4], Linda Petzold[5,6]

1 Department of Chemical Engineering, University of California, Santa Barbara, California; United States of America
2 Department of Statistics and Applied Probability, University of California, Santa Barbara, California; United States of America
3 Department of Geography, University of California, Santa Barbara, California; United States of America
4 Science for Life Laboratory, Department of Information Technology, SE-751 05, Uppsala University, Uppsala, Sweden
5 Department of Mechanical Engineering, University of California, Santa Barbara, California; United States of America
6 Department of Computer Science, University of California, Santa Barbara, California; United States of America

* colbyfronk@ucsb.edu

Supplementary Information

S1 Bayesian Linear Regression Derivation

Prior Distribution:

$$w \sim \mathcal{N}(0, S) \tag{1}$$

Likelihood:

$$t|x, w \sim \mathcal{N}(w^T \Phi(x), \sigma^2) \tag{2}$$

Deriving the Posterior Distribution assuming fixed S and σ^2:

$$\begin{aligned}
log(p(w|D)) &= log(p(w)) + log(p(D|w)) + const && (3)\\
&= -\frac{1}{2} w^T S^{-1} w - \frac{1}{2\sigma^2} ||\phi w - t||^2 + const && (4)\\
&= -\frac{1}{2} w^T S^{-1} w - \frac{1}{2\sigma^2} (w^T \phi^T \phi w - 2t^T \phi w + t^T t) + const && (5)\\
&= -\frac{1}{2} (w - \mu)^T \Sigma^{-1} (w - \mu) + const && (6)
\end{aligned}$$

Where μ and Σ are given by:

$$\begin{aligned}
\mu &= \sigma^{-2} \Sigma \phi^T t && (7)\\
\Sigma^{-1} &= \Sigma^{-2} \phi^T \phi + S^{-1} && (8)
\end{aligned}$$

This is a multivariate Gaussian distribution with:

$$w|D \sim \mathcal{N}(\mu, \Sigma) \tag{9}$$
